# Design and Vibration Suppression Control of a Modular Elastic Joint

**DOI:** 10.3390/s18061869

**Published:** 2018-06-07

**Authors:** Hong Liu, Shipeng Cui, Yiwei Liu, Yi Ren, Yongjun Sun

**Affiliations:** State Key Laboratory of Robotics and System, Harbin Institute of Technology, West Dazhi Street, Harbin 150001, China; hong.liu@hit.edu.cn (H.L.); cuishipeng@hit.edu.cn (S.C.); yipor@stu.hit.edu.cn (Y.R.); sunyongjun@hit.edu.cn (Y.S.)

**Keywords:** elastic joint, customized torsional spring, fail-safe brake, resonance ratio control, disturbance observer

## Abstract

In this paper, a novel mechatronic design philosophy is introduced to develop a compact modular rotary elastic joint for a humanoid manipulator. The designed elastic joint is mainly composed of a brushless direct current (DC) motor, harmonic reducer, customized torsional spring, and fail-safe brake. The customized spring considerably reduces the volume of the elastic joint and facilitates the construction of a humanoid manipulator which employs this joint. The large central hole along the joint axis brings convenience for cabling and the fail-safe brake can guarantee safety when the power is off. In order to reduce the computational burden on the central controller and simplify system maintenance, an expandable electrical system, which has a double-layer control structure, is introduced. Furthermore, a robust position controller for the elastic joint is proposed and interpreted in detail. Vibration of the elastic joint is suppressed by means of resonance ratio control (RRC). In this method, the ratio between the resonant and anti-resonant frequency can be arbitrarily designated according to the feedback of the nominal spring torsion. Instead of using an expensive torque sensor, the spring torque can be obtained by calculating the product of spring stiffness and deformation, due to the high linearity of the customized spring. In addition, to improve the system robustness, a motor-side disturbance observer (DOb) and an arm-side DOb are employed to estimate and compensate for external disturbances and system uncertainties, such as model variation, friction, and unknown external load. Validity of the DOb-based RRC is demonstrated in the simulation results. Experimental results show the performance of the modular elastic joint and the viability of the proposed controller further.

## 1. Introduction

Robots, especially those working in industrial environments, have been generally designed according to the principle “rigidity by design, safety by sensors and control”. The safety to interact with rigid environments is usually guaranteed by means of active compliance control, i.e., position/force or impedance/admittance control [1,2,3]. However, the delays existing in the software system make the rigid actuator unsafe during sudden impacts and is incapable of handling contact transients. To overcome these difficulties, series elastic actuators (SEA) are proposed as a simple and effective solution to realize inherent compliance [4]. Unlike a rigid actuator, a SEA contains an elastic element in series, which introduces low mechanical output impedance, passive mechanical energy storage, and increases peak power output. Moreover, the inherent compliance brought by the elastic element can filter impact, protect drivetrains, and provide additional time for active controllers to regulate the impedance of the actuator.

SEAs have been successfully applied in numerous scenarios for more than 20 years. A prismatic SEA, using a ball-screw mechanism for speed reduction, was designed in reference [5]. This mechanism is highly efficient and has a high tolerance for impact loads. However, the main drawback of using a ball-screw mechanism in a SEA is that an extra output mechanism is needed for converting translational motion to rotation. In reference [6], a new compact soft actuation unit intended to be used in multi-DOF and small scale robotic systems was introduced. Although the designed actuator shows high integration density and wide passive compliance, the torsional spring designed based on an arrangement of linear springs is still too thick to increase the size of the actuator and suffers from nonlinear stiffness. Cummings et al. [7] designed a compact modular rotary SEA, which can meet the requirements of a wide range of applications. A customized torsional spring designed for the NASA Robonaut 2 platform was used in this SEA. However, the employment of planetary gearhead made the SEA unsuitable for a manipulator, despite a novel sensor geometry being presented for mitigating the negative impact of backlash. Moreover, SEAs are also widely employed in bipedal walking robots [8], robotic devices for rehabilitation [9,10], and wearable robotics for assistance [11]. Those SEAs also suffer from large size, non-modularity, and mechanical design complexity. Recently, variable stiffness actuators (VSAs) [12,13] have been introduced to maximize benefits of SEA with respect to safety during interaction, as well as to improve the energy efficiency of the robotic system. VSAs typically employ two motors, which respectively control the compliance and the equilibrium position of the actuated joint. The introduction of an extra motor to achieve the functionality of the stiffness adjustment may lead to the increase of complexity, size, and the total weight of the actuation system.

Another problem is that introduction of the elastic element reduces the actuator bandwidth and makes position control difficult [14]. Moreover, it is prone to vibrate, which deteriorates system stability and requires extra power for position control [15]. Several approaches have been proposed to control the position of elastic-joint robots. Tomei [16] proved that a simple proportional-differential (PD) controller can globally stabilize about a reference position. Kim et al. [17] proposed a robust PD control scheme for flexible-joint robots based on a DOb which was only applied to the motor-side dynamics. Jin et al. [18] designed an adaptive tracking controller using a time-delay estimation technique. While his approach was to achieve accurate motor-side position before the elastic element and let the arm side show passive compliant behaviors. Additionally, many vibration suppression approaches for elastic joints or elastic-joint robots have been proposed and explicit analyses have been presented. As an effective vibration suppression control method, joint damping controllers have been reported in the literature. Well-known approaches such as feedback linearization [19], model-based state-feedback controllers [20], learning control schemes [21], or linear-quadratic regulators [22], have been developed. These schemes are highly effective and show great performance. But, a limitation of these approaches is their dependence on an accurate mathematical model. In order to solve this problem, Petit et al. [23] proposed a model-free damping control approach, which utilized the joint elasticities to convert kinetic energy into elastic energy to achieve vibration suppression. However, this control approach was employed mainly to deal with the vibration introduced by disturbances. Besides, a number of feedforward control techniques have been applied to robots with elastic joints. In reference [24], a command shaping method has been used for flexible joint to reduce vibration. The same goal has been achieved for a two-inertia system [25], in which a robust control scheme combining backstepping technique and partial DOb has been proposed. This controller also included a reference generator, which provided a reference state and a feedforward input signal. Although it plays an important role in vibration suppression, feedforward control reaches a compromise between rapidity and shaper robustness for positive input shapers and smoothly shaped reference commands [26]. Furthermore, controllers of a prismatic SEA for force and position tracking based on combinations of PID, model-based, and DOb structures were presented in reference [5]. It is noted that actuator resonance is suppressed not only by the DOb but also by the joint structure in which the spring is placed between the motor housing and the chassis ground.

In this paper, a novel compact, modular rotary elastic joint is designed in detail. The designed elastic joint is evolved from the traditional rigid joint in which the expensive torque sensor is replaced by a torsional spring. This design philosophy greatly reduces the design cycle and cost. As to the mechanical system, the joint adopts a new customized torsional spring as an elastic element. The customized torsional spring is optimized using Finite Element Method (FEM) simulations to satisfy the specific performance requirements. A large central hole along the joint axis is introduced to ease cabling. In order to maintain position when an outage happens, a fail-safe brake is designed. In the electrical system, a double-layer control structure, comprising a central controller and a joint controller, is employed to reduce the computational burden and simplify system maintenance. Besides, the double-layer control structure of electrical system is suitable for constructing a humanoid manipulator in future. The central controller is mainly responsible for the implementation of a high-level control algorithm, and joint controller is responsible for sensor information processing, motor and fail-safe brake control. Data communication between central controller and joint controller is achieved by a Point-to-Point High Speed Serial Communication (PPSeCo) protocol. All the configurations above make the designed elastic joint light weight, small volume, and suitable as a module for a humanoid manipulator. In addition, a high-performance robust position controller is proposed for the elastic joint. Vibration is suppressed by using RRC, of which the control gains are determined theoretically. In order to improve the system robustness, a motor-side DOb and arm-side DOb are employed to deal with external disturbances and system uncertainties, such as model variation, friction, and load. Simulation and experimental results are given to show the validity of the DOb-based RRC.

This paper highlights research in design and vibration suppression control of a modular elastic joint. Our contributions include: (1) A mechanical design of the elastic joint which is more compact and lightweight than previous SEA designs; (2) a design of the non-uniform spiral spring employing only one profile variable parameter, which greatly reduces the complexity of optimization process; and (3) precise point-to-point position control with which vibration is effectively suppressed. This paper is organized as follows. In Section 2, the mechanical and electrical designs of the elastic joint are elaborated. Section 3 proposed a DOb-based RRC with a detailed explanation and analysis. In Section 4, position control simulations and experimental results are given to validate the efficacy of the proposed control scheme. Section 5 presents the conclusions.

## 2. Elastic Joint Design

For a humanoid manipulator, a modular design can strengthen interchangeability of the parts and eliminate structural differences between the joints. In terms of the configuration and workload, a humanoid manipulator can be established by two types of modular joints, namely shoulder/elbow joint and wrist joint. They are identical in the mechanical structure and the electrical system, except for the selection of some standard components. In this paper, an elastic joint in shoulder/elbow is designed. Design indexes and actual values are listed in Table 1.

### 2.1. Mechanical Design

**Figure 1:**
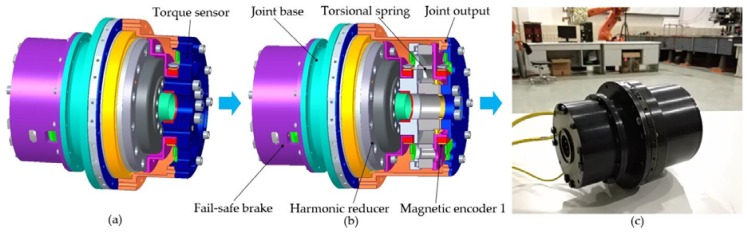
The design process of the elastic joint. (**a**) Traditional rigid joint with torque sensor; (**b**) Cross section view of the prototype elastic joint; (**c**) Physical prototype of the elastic joint.

The ideal objective of the elastic joint design is to achieve a compact and modular mechanical architecture. In order to reduce design circle and cost, the designed elastic joint is evolved from the traditional rigid joint, which replaces the expensive torque sensor with the torsional spring. The design diagram of the elastic joint is shown in Figure 1b. The final assembly of the elastic joint consists of a brushless DC motor, harmonic reducer, customized torsional spring, bearings, magnetic encoders, fail-safe brake, etc. The motor stator and circular spline of the harmonic reducer are fixed on the joint base. The motor rotor drives the wave generator through the motor shaft and the flexspline of the harmonic reducer drives the joint output through the torsional spring. The rotation disk and shell of the fail-safe brake are fixed on the motor shaft and the shell of the joint respectively. All housing parts are made of aluminum alloy 7A09 for the purpose of weight reduction.

- Motor components: A brushless DC motor 72-L designed by Harbin Institute of Technology (HIT) is selected. The rotor has large center hole and the maximum diameter of the stator is 72 mm. Its rated torque and maximum torque are respectively 0.71 Nm and 0.85 Nm respectively.
- Reducer components: A harmonic reducer CSD-25 of Harmonic Drive Company is selected. Its single-stage reduction ratio is 160 and the corresponding peak torque is 123 Nm.
- Customized torsional spring: To minimize the volume of the joint, a customized torsional spring is designed. Compared to the existing torsional springs, this spring has the characteristics of small volume and high linearity between applied torque and deformation. See Section 2.3 for more details.
- Bearings: A deep groove ball bearing is used to guarantee that the two rings of the torsional spring rotate coaxially. Two deep groove ball bearings are used to support the motor shaft. By replacing a pair of traditional angular contact ball bearings, a cross roller bearing THK-RA8008C, which supports the joint output, can make the joint more compact.
- Magnetic encoders: All position information is measured by three 16-bit absolute magnetic rotary encoders designed by HIT. Motor position, motor position after reduction, and spring deformation β can be measured respectively. The motor-side position θ, used in the control system is calculated by means of sensor data fusion [27] between motor position and motor position after reduction. The arm-side position *q* is calculated by the difference between the motor-side position and the spring deformation, i.e., q=θ−β. The magnetic encoder consists of two inductors, i.e., a code disk and a signal processing part. It possesses small-size, light, robust, and easy-to-integrate properties.
- Fail-safe brake: In order to maintain current position when an outage happens, the elastic joint integrates with the fail-safe brake, which is mainly composed of a rotation disk, wear-resisting friction disks, a pressing disk, an electromagnet, springs, and a shell. Eight wear-resisting friction disks are evenly distributed on the rotation disk. The force generated by the electromagnet separates the pressing disk against the spring force from the friction disks when the power is on. When the power is off, the spring force presses the pressing disk on the friction disks, which makes the motor shaft stop. The fail-safe brake designed in this paper has the advantages of low energy consumption and fast braking.

Employments of standard (CSD-25, THK-RA8008C) and customized components (brushless DC motor, magnetic encoders, torsional spring) make the designed elastic joint more compact. The large central hole diameter of 17 mm along the joint axis brings convenience for cabling. The integrated fail-safe brake can stop the joint and guarantee safety when the power is off. The designed elastic joint only uses three 16-bits absolute magnetic rotary encoders for sensor information, rather than expensive sensors such as a torque sensor. Compared to the existing elastic joints, the designed elastic joint has the characteristics of light-weight, small volume and high energy, which is suitable for a module for a humanoid manipulator. Further, the designed joint is able to achieve full rotation and has little hysteresis.

### 2.2. Electrical System Architecture

**Figure 2:**
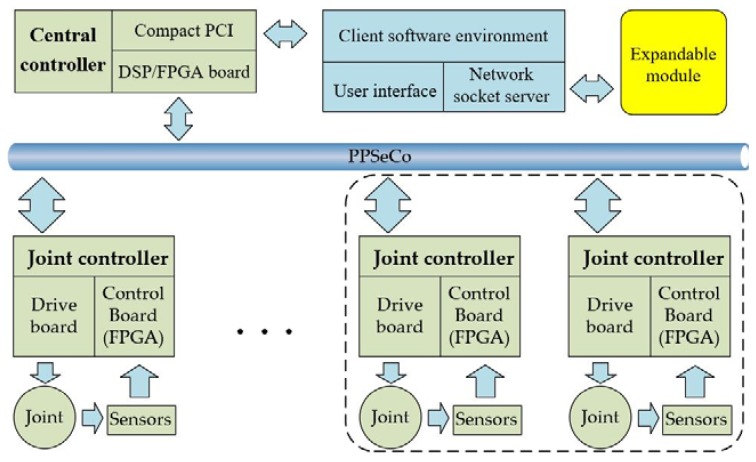
Electrical system diagram of the elastic joint.

In this section, an expandable electrical system architecture is proposed as shown in Figure 2. The dashed part represents other joints, which can together constitute a humanoid manipulator. The electrical system adopts double-layer control structure, which mainly consists of a central controller and a joint controller. Some key factors of this scheme are taken into account. First of all, the double-layer control structure can reduce the computational burden and simplify system maintenance compared to a single controller. Another reason for the choice of double-layer control structure is that it facilitates building a humanoid manipulator in the future.

The main tasks performed by the central controller are motion planning of elastic joint or manipulator, implementation of control algorithm, and providing interface with host computer. The joint controller is responsible for sensor information processing, motor and fail-safe brake control, etc. In order to increase the transmission reliability and speed of data communication, reduce cabling and noise in sensor signals, a PPSeCo protocol proposed by our lab is employed here for the data communication between central controller and joint controller [28]. All communication programs are written in VHDL and run in FPGAs. The frequency of system is 500 Hz determined by the software interrupt.

The hardware of the central controller is a Compact PCI-based DSP/FPGA board. The main processor of the central controller is a TI floating-point DSP TMS320C6713 with a maximum 1,350 MFLOPS. The DSP is an excellent choice to easily realize complex control algorithm and fast computation because of its high performance floating-point capabilities. Furthermore, the DSP provides a series of peripherals, which make it easy for user to access and extend the hardware resources. Serial signals from the joint controller are converted to parallel signals and transmitted to the DSP via the parallel interface by the FPGA, and vice versa. The DSP/FPGA board and host computer are packed into a cabinet based on a Compact PCI structure, as well as a DC/DC converter which provides power for joint motor, sensors, and fail-safe brake.

Joint controller is composed of a driver board, a control board, and a connecting board. The control board adopts the Cyclone IV series FPGA produced by Altera Company (San Jose, CA, USA) as the microprocessor to realize the control of the motor and the fail-safe brake, sensor information processing, and high-speed serial communications with the central controller. The drive board receives the commands from the control board to drive the motor and the fail-safe brake. In order to reduce the modular joint’s weight and volume, the joint controller is placed outside the joint and is intended to be placed inside the link in the future.

### 2.3. Spring Design

Being a fundamental component of the SEA, the elastic element has great influence on the size and mechanical properties of the joint. Thus, the design of the torsional spring is the key for the rotary SEA. Generally, there are two forms of torsional spring design in the literature. The first form [6,29] is usually based on an arrangement of linear springs, which leads to a larger volume. The second form adopts a customized design to minimize the volume and improve performance of the torsional spring. A systematic approach to the design of a monolithic torsional elastic module has been presented in reference [30]. The designed elastic module shows a good linearity characteristic between torque and angle. By arranging a number of identical springs in series or in parallel, it is possible to render different torque vs. angle characteristics, in order to match the special application requirements. Also employing the customized form, various torsional springs [7,10,31] have been designed and used in SEAs. Those torsional springs have the advantages of light-weight and small volume. In the design of the customized torsional spring, the first step is choosing a set of topologies. Commonly there are three topologies of the customized the torsional spring:

- Groups of symmetrical flexible beams are placed between the inner and outer ring of the spring. Due to the limited space, flexible beams in such springs are usually thin and thus can only withstand relatively low torque [31].
- Uniform spiral beams. The typical representation is an Archimedean spiral torsional spring. Although the spring employing this topology has a good ability to withstand high torque, hysteresis and backlash in its structure may deteriorate the dynamic performance [10].
- Non-uniform spiral beams. The spring employing this topology can withstand high torque. However, the profile of the non-uniform spiral beams, which is depicted by a multi-segment curve is difficult to construct [7]. Furthermore, this kind of spring has more profile parameters, which leads to greater complexity of the optimization.

**Figure 3:**
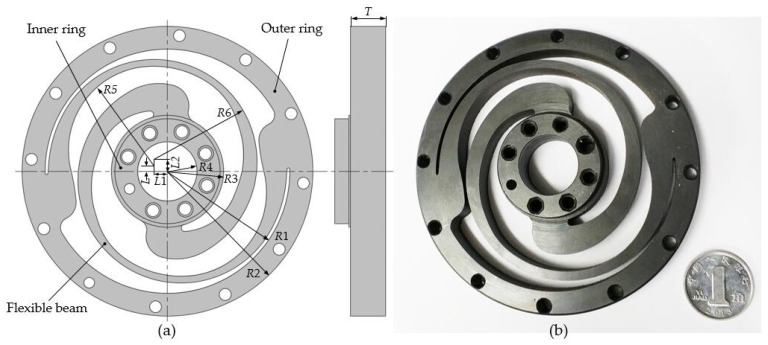
The designed torsional spring. (**a**) Schematic perspective view of the customized torsional spring; (**b**) Physical model of the customized torsional spring.

In order to realize small volume and high-performance index of the output torque, a customized torsional spring which has non-uniform spiral beams is designed in this paper. Different from the previous torsional spring, the spring designed in this paper employs eccentric-arcs to build non-uniform spiral beams, which requires only one profile variable parameter. Figure 3a shows a schematic perspective view of the customized torsional spring. It is composed of two rings interconnected by a pair of non-uniform spiral beams, which are outlined by eccentric-arcs *R*5 and *R*6 with different radii. *R*5 and *R*6 are equal to 50 mm and 58 mm, respectively. The arc lengths of *R*5 and *R*6 are about 3π/2. The values of *R*1, *R*2, *R*3, and *R*4 are constant (70 mm, 83 mm, 32 mm, and 17 mm) and constrained by the elastic joint dimensions. To simplify the design, the values of *L*1 and *L*2 are set to be constant. The corresponding design variables are, respectively, vertical eccentric distance *L* and spring thickness *T*. The thickness of the flexible beam changes with different *L*.

Flexible beams generate elastic deformation to transfer the torque and absorb the impact when two rings rotate relatively. Spring steel 50CrVA is selected as the spring material of which the Young’s modulus and yield strength are 205 GPa and 1,320 MPa, respectively. The stress distribution and deformation of the customized torsional spring was analyzed by FEM to ensure that the maximum stress is less than the yield strength of the material. The theoretical spring stiffness is computed by the ratio between the applied torque and the corresponding angular deformation obtained in the simulation.

**Figure 4:**
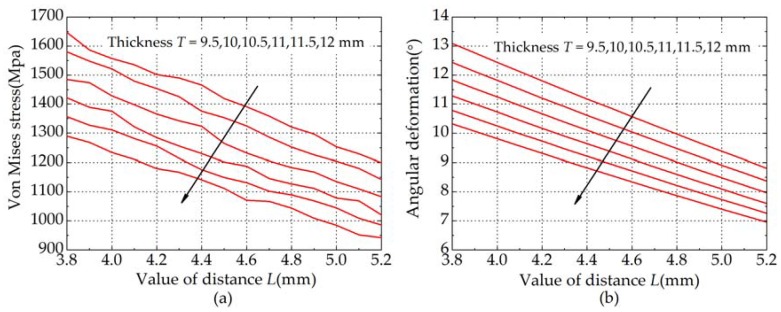
Results of the methodology adopted. The arrows indicate the direction of increasing spring thickness. (**a**) Geometry parameter *L* vs. Von Mises stress; (**b**) Geometry parameter *L* vs. Angular deformation of the spring.

The spring is responsible for almost all the passive compliance of the elastic joint and affects the dynamics of the elastic joint, the torque sensing range and resolution. Thus, proper choice of the spring stiffness is critical. However, choosing the optimal spring stiffness $K_{sdesire}$ for an elastic joint of humanoid manipulator is still under investigation and controversial discussion [32]. Some indications lead to a value of about 350 Nm/rad in the elbow [33]. Then, the maximum angular deformation of the joint $\beta_{\max}$ can be calculated as follows.

(1) $$\beta_{\max}=\frac{\tau_{\max}}{K_{sdesire}}=\frac{60\;\mathrm{Nm}}{350\;\mathrm{Nm}/\mathrm{rad}}=0.171\;\mathrm{rad},$$

where $\tau_{\max}$ is the max elastic output torque of the elastic joint. The methodology to determine the geometry parameters of the spring is presented as follows. The value of distance *L* is varied from 3.8 mm to 5.2 mm with a step of 0.1 mm for each thickness *T* from 9.5 mm to 12 mm with a step of 0.5 mm. Figure 4 shows the results of the adopted methodology. It can be concluded that spring becomes more stiff and robust when *T* is thicker. From Figure 4a, it can be observed that Von Mises stress roughly decreases as the variable *L* increases. As shown in Figure 4b, the relationship between variable *L* and angular deformation at different thicknesses of the spring can be characterized by a linear approximation.

**Figure 5:**
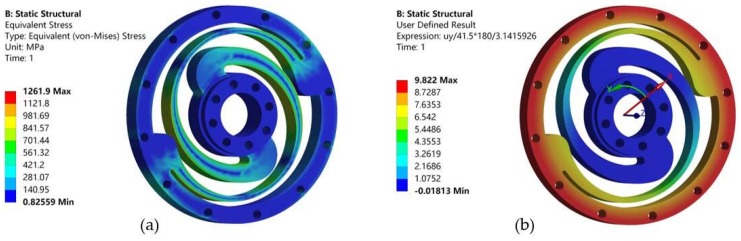
Analysis results of spring by FEM. (**a**) Static simulation for stress distribution; (**b**) Static simulation for angular deformation.

When the geometry parameters *L* and *T* are, respectively, 4.5 mm and 10.5 mm, the spring stiffness obtained in the simulation is approximately 350 Nm/rad and the torsional spring is subjected to minimal stress. Correspondingly, the maximum value of Von Mises stress obtained in the simulation is 1261.9 MPa as shown in Figure 5. The customized torsional spring, shown in Figure 3b, is manufactured by using the Wire Electrical Discharge Machining (WEDM) process.

**Figure 6:**
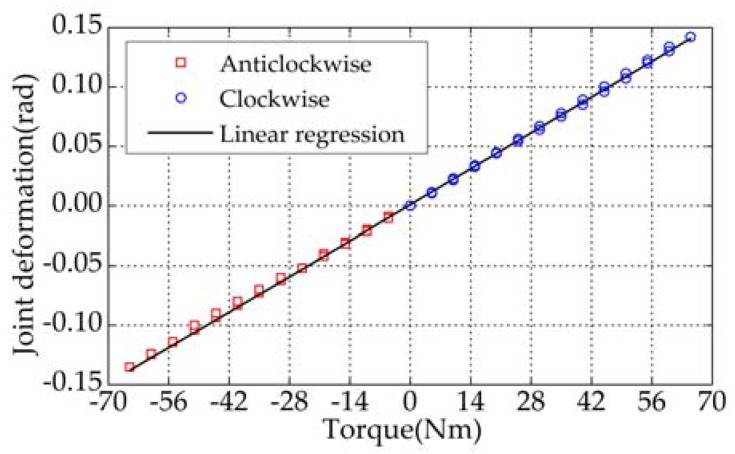
Characteristics of the customized torsion spring.

Actual stiffness of the designed spring should be calibrated. In this paper, it can be achieved by measuring joint stiffness by virtue of loading experiments. Loading and unloading are implemented on the flange which is mounted on the joint output. The torque exerted on the joint output can be calculated. Figure 6 shows that the calculated torque and the corresponding angular deformation of the elastic joint measured by the magnetic encoder 1. As shown in Figure 6, there is little hysteresis with loading and unloading on the joint. A linear regression was performed in both directions and the corresponding value of the joint stiffness can be calculated as 464.2 Nm/rad. The value of the joint stiffness, experimentally determined, is approximately 32.6% larger than the spring stiffness obtained by finite element analysis. This discrepancy probably arises in the imperfection of model used in the analysis and the different properties between the actual material after thermal treatment and the nominal counterparts. Besides, measurement errors of the sensor, the load and the arm may have a great influence on the experimental results.

## 3. Position Controller Design

### 3.1. Dynamic Model

As shown in Figure 1, the motor side is connected to the arm side with the torsional spring of which its elasticity easily leads to resonance in the system. The dynamic equation of the elastic joint can be described by:

(2) $$M\ddot{\theta }+f_{m}+\tau_{s}=\tau_{u};$$

(3) $$J\ddot{q}+f_{l}+g(q)=\tau_{s};$$

(4) $$\tau_{s}=K_{s}(\theta -q),$$

where M, J are the inertias of motor side and arm side. $f_{m}$ and $f_{l}$ are viscous and coulomb frictions of motor side and arm side respectively. $\tau_{u}$ is the motor-side input torque and $\tau_{s}$ is the spring torque due to the joint deformation specified in Equation (4). g(q) is the gravitational torque and $K_{s}$ denotes the joint stiffness.

**Figure 7:**
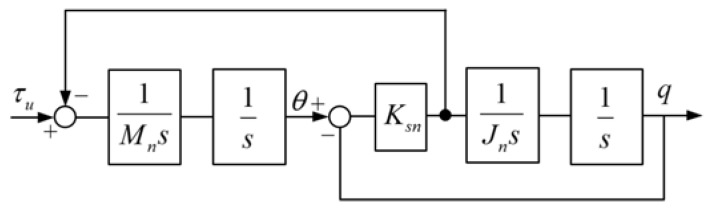
Model of two-mass resonant system.

In practice, real model parameters cannot be achieved, i.e., we can only obtain nominal model parameters. So, the dynamic model of the elastic joint can be reformulated as follows:

(5) $$M_{n}\ddot{\theta }+D_{m}+\tau_{sn}=\tau_{u};$$

(6) $$J_{n}\ddot{q}+D_{l}=\tau_{sn},$$

(7) $$\tau_{sn}=K_{sn}(\theta -q),$$

where $M_{n}$ and $J_{n}$ are nominal inertia of motor side and arm side respectively. $D_{m}$ and $D_{l}$ denote lumped terms including all nonlinearities, uncertainties, and external disturbances, which can be defined as:

(8) $$D_{m}=(M-M_{n})\ddot{\theta }+f_{m}+(\tau_{s}-\tau_{sn});$$

(9) $$D_{l}=(J-J_{n})\ddot{q}+f_{l}+g(q)-(\tau_{s}-\tau_{sn}),$$

when $D_{m}$ and $D_{l}$ are neglected, joint dynamic equation can be simplified into two-mass resonant system and the block diagram is shown in Figure 7.

From Figure 7, the transfer function from the motor-side torque $\tau_{u}$ to the motor-side position θ and the transfer function from the motor-side torque $\tau_{u}$ to the arm-side position q can be expressed as follows:

(10) $$\frac{\theta }{\tau_{u}}=\frac{\frac{1}{M_{n}}(s^{2}+\frac{K_{sn}}{J_{n}})}{s^{4}+(\frac{K_{sn}}{J_{n}}+\frac{K_{sn}}{M_{n}})s^{2}};$$

(11) $$\frac{q}{\tau_{u}}=\frac{\frac{K_{sn}}{M_{n}J_{n}}}{s^{4}+(\frac{K_{sn}}{J_{n}}+\frac{K_{sn}}{M_{n}})s^{2}}.$$

Then, the anti-resonant frequency $\omega_{ar}$ in the arm side, which represents the natural frequency of the elastic joint and the resonant frequency, $\omega_{r}$, in the motor side can be described as follows:

(12) $$\omega_{ar}=\sqrt{\frac{K_{sn}}{J_{n}}};$$

(13) $$\omega_{r}=\sqrt{\frac{K_{sn}}{J_{n}}+\frac{K_{sn}}{M_{n}}}.$$

**Figure 8:**
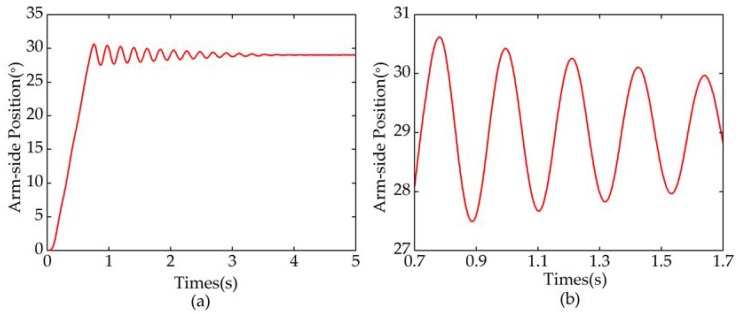
Results of the arm-side free oscillation experiment. (**a**) Arm-side position; (**b**) Magnified view of (**a**).

The characteristics of the two-mass resonant system can be determined if model parameters $K_{sn}$, $M_{n}$ and $J_{n}$ are known. Joint stiffness ($K_{sn}=464.2\;\mathrm{Nm}/\mathrm{rad}$) can be obtained by the experiment stated in Section 2.3. The inertia of motor side $M_{n}$ can be calculated by three-dimensional model of the elastic joint established in PRO/ENGINEER. The inertia of motor side is mainly determined by the rotor, motor shaft. and wave generator. Multiplying the sum of these inertia values by square of the reduction ratio *N*, the inertia of motor side can be obtained, namely, $M_{n}=M_{\mathrm{total}}\cdot N^{2}=1.567\;\mathrm{Kg}\cdot m^{2}$. According to Equation (12), the motor-side inertia $J_{n}$ is equal to $K_{sn}/\omega_{ar}^{2}$. Due to the low arm-side friction, $K_{sn}$ is close to $K_{s}$. In addition, the anti-resonant frequency $\omega_{ar}$ can be obtained by a free oscillation experiment, in which the motor is controlled to rotate to a certain position and then locked by the brake. Figure 8 shows the arm-side position in free oscillation experiment, from which we can know that the oscillation period $T_{n}$ is about 0.214 s. So, the inertia of motor side $J_{n}$ can be calculated as 0.528 Kg·m^2^.

### 3.2. Disturbance Observer

**Figure 9:**
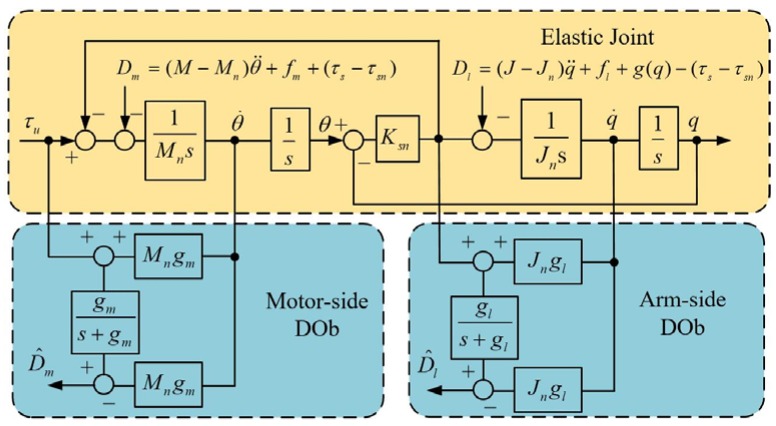
Block diagram of motor-side DOb and arm-side DOb.

Robustness is essential to design a high-performance motion control system in practice. A DOb. which estimates external disturbances and system uncertainties, such as external load, friction, and inertia variation is widely used to achieve the robustness of motion control systems due to its simplicity and efficiency [34,35]. In this paper, a motor-side DOb and an arm-side DOb are both employed, as shown in Figure 9. Here, the disturbance torques on the motor side and arm side are defined in Equations (8) and (9), respectively. The outputs of the DObs, which can be described as the following two equations, are the estimation values of external disturbance:

(14) $${\hat{D}}_{m}=\frac{g_{m}}{s+g_{m}}(D_{m}+\tau_{sn});$$

(15) $${\hat{D}}_{l}=\frac{g_{l}}{s+g_{l}}D_{l},$$

where $g_{m}$ and $g_{l}$ are the bandwidths of motor-side and arm-side DOb respectively. The bandwidth of DOb is directly related to the robustness of the motion control system. The higher the bandwidth of DOb is, the more robustness can be expected. It should be noted that the actual bandwidth of DOb is limited by practical constraints such as noise, sampling time, etc. [36].

### 3.3. Resonance Ratio Control

**Figure 10:**
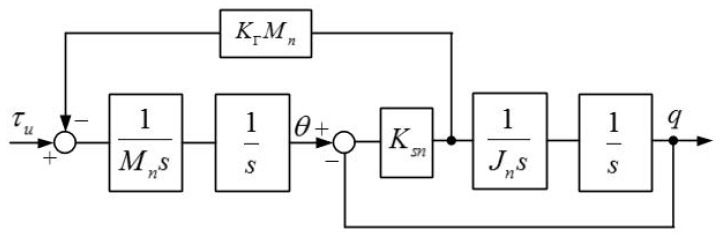
Spring torque feedback multiplying *K*_Γ_*M_n_*.

As a novel control method, the resonance ratio control first proposed by Yuki et al. [37] can suppress the vibration for the position control in two-mass resonant system. If the spring torque multiplying $K_{\Gamma }M_{n}$ is feedback to the two-mass resonant system, the system block diagram can be transformed from Figure 7 into Figure 10. When $K_{\Gamma }=1/M_{n}$, the system shown in Figure 10 has equivalent performance of the uncontrolled plant shown in Figure 7.

Then, the anti-resonant frequency $\omega_{ar}$ and the resonant frequency $\omega_{a}$ of the new system can be described as follows:

(16) $$\omega_{ar}=\sqrt{\frac{K_{sn}}{J_{n}}};$$

(17) $$\omega_{r}=\sqrt{\frac{K_{sn}}{J_{n}}(1+K_{\Gamma }J_{n})}=K\omega_{ar},$$

where K is called “Resonance Ratio” and defined as the following equation:

(18) $$K=\sqrt{\left(1+K_{\Gamma }J_{n}\right)}.$$

**Figure 11:**
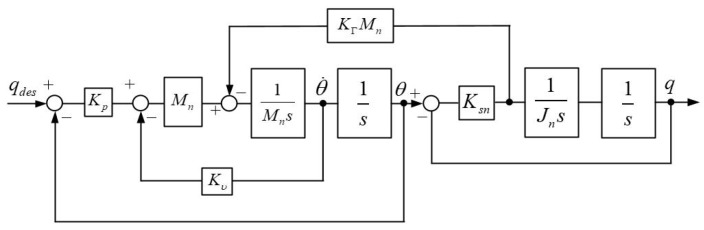
PD control of the two-mass resonant system.

It is apparent from the definition in Equation (18) that the resonance ratio is a monotone increasing function of the spring torque feedback gain $K_{\Gamma }$. This means that the resonance frequency and the resonance ratio of the system can be tuned to an arbitrary value. Intuitively, the role of $K_{\Gamma }$ is to depict to what extent the motor side is affected by the spring torque and represent the sensitivity of the motor side against the spring torque, i.e., the arm side. A PD control in the motor side is constructed as shown in Figure 11. This controller consists of a proportional gain $K_{p}$, a differential gain $K_{\upsilon }$, and a spring torque feedback gain $K_{\Gamma }$.

From Figure 11, the transfer functions from $q_{des}$ to θ and q are given as follows, respectively:

(19) $$\theta =\frac{K_{p}(s^{2}+\omega_{ar}^{2})}{s^{4}+K_{\upsilon }s^{3}+(K_{p}+\omega_{r}^{2})s^{2}+K_{\upsilon }\omega_{ar}^{2}s+K_{p}\omega_{ar}^{2}}q_{des};$$

(20) $$q=\frac{K_{p}\omega_{ar}^{2}}{s^{4}+K_{\upsilon }s^{3}+(K_{p}+\omega_{r}^{2})s^{2}+K_{\upsilon }\omega_{ar}^{2}s+K_{p}\omega_{ar}^{2}}q_{des}.$$

In the position RRC, it is shown that the vibration can be suppressed by controlling the ratio between resonant and anti-resonant frequencies via spring torque feedback gain $K_{\Gamma }$. Expressions of each control gain are given as follows [37]:

(21) $$K=\frac{\omega_{r}}{\omega_{ar}}=\sqrt{5};$$

(22) $$K_{p}=\omega_{ar}^{2},$$

(23) $$K_{\upsilon }=4\omega_{ar};$$

(24) $$K_{\Gamma }=4/J_{n}.$$

**Figure 12:**
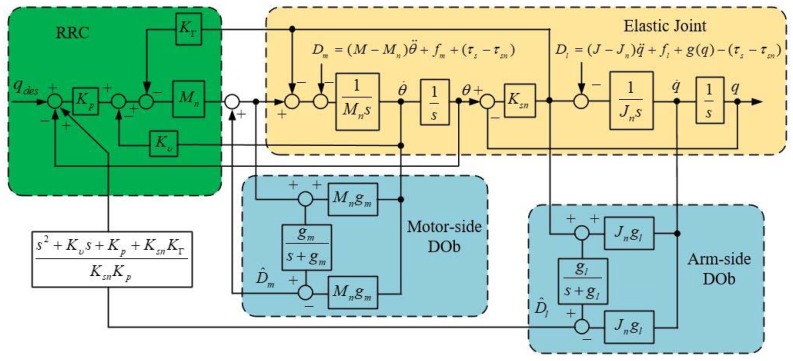
The position resonance ratio control based on arm-side and motor-side DOb.

In order to improve the robustness of the position control system, a motor-side DOb and an arm-side DOb explained in Section 3.2 are employed here, as shown in Figure 12.

Robust position control of the motor side is achieved by feedback to the inner-loop DOb. The arm-side disturbance, estimated by the arm-side DOb, is compensated by feedback through the inverse system. The inverse system can be calculated by the transfer function from $q_{des}$ to q as shown in Equation (20) and from $D_{l}$ to q as follows:

(25) $$\frac{q}{D_{l}}=\frac{(s^{2}+K_{\upsilon }s+K_{p}+K_{sn}K_{\Gamma })/J_{n}}{s^{4}+K_{\upsilon }s^{3}+(K_{p}+\omega_{r}^{2})s^{2}+K_{\upsilon }\omega_{ar}^{2}s+K_{p}\omega_{ar}^{2}}q_{des}.$$

Then, the inverse system can be represented by the following equation:

(26) $$\frac{q_{des}}{D_{l}}=\frac{s^{2}+K_{\upsilon }s+K_{p}+K_{sn}K_{\Gamma }}{K_{p}K_{sn}}.$$

For the sake of simplicity, the following approximate transfer function is generally used with an arm-side DOb:

(27) $$\frac{q_{des}}{D_{l}}=\frac{s^{2}+K_{\upsilon }s+K_{p}+K_{sn}K_{\Gamma }}{K_{p}K_{sn}}≅\frac{K_{p}+K_{sn}K_{\Gamma }}{K_{p}K_{sn}}.$$

It should be noted that, only constant disturbances can be suppressed if the higher order derivatives are neglected in Equation (27).

## 4. Simulation and Experimental Results

Firstly, numerical simulation is given to confirm the validity of the proposed control approach. In the simulation, the sampling period of simulation system was 2 ms and the real model parameters were set to 20% larger than the nominal counterparts. The arm-side inertia varies a lot with the load in practice, so the real arm-side inertia was also set to double for comparison. The load and the arm length were set to 4 Kg and 0.3 m respectively. Coulomb friction and viscous friction coefficients of the motor side as well as the arm side were 0.05 Nm and 0.1 Nm·s/rad respectively. Controller gains, nominal model parameters of the elastic joint obtained in Section 3.1 and the bandwidths of DObs are listed in Table 2.

**Figure 13:**
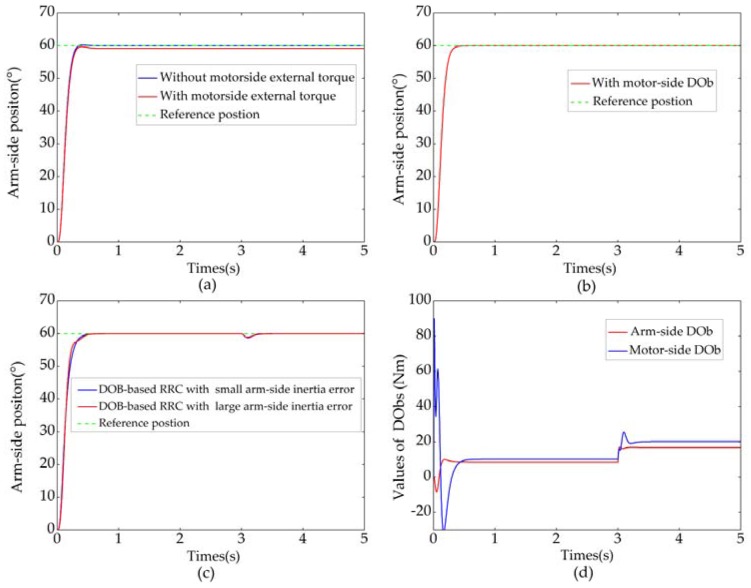
Simulation results of position RRC. (**a**) Position RRC without DObs; (**b**) Position RRC with motor-side Dob; (**c**) Position RRC with DObs; (**d**) Outputs of DObs.

Figure 13a–d shows the simulation results of position control when a step reference input ($q_{des}=60{}^{\circ}$) was applied. If the arm-side disturbance was neglected, the position RRC without motor-side DOb is shown in Figure 13a. Due to large $K_{p}$ and small motor-side disturbance, there was almost no discrepancy between q and $q_{des}$. It is noteworthy that arm-side position responded rapidly, but there was a small overshoot. If external torque of 20 Nm was deliberately applied to the motor side, the steady-state error appeared. As it is shown in Figure 13b, the steady-state error caused by motor-side disturbance can be eliminated by virtue of the proposed motor-side DOb. It is clear from the figure that the motor-side DOb improved not only the steady-state performance but also the transient response of the system. Figure 13c shows the result of position RRC with DObs. An arm-side external torque of 10 Nm was applied at 3 s. After a short adjustment period, the arm-side position then kept following the step reference input quickly and smoothly. The robustness of the system was improved by using the arm-side DOb. Moreover, the proposed DOb-based RRC was robust against much larger disturbance on the nominal model, as shown in Figure 13c. The outputs of the arm-side DOb and motor-side DOb are shown in Figure 13d. Besides system uncertainties such as model variation, friction, and load, the estimated value of the arm-side disturbance contained external torque. Figure 13a–c clearly shows that the vibration can be suppressed by using the proposed RRC method. Simulation results show the validity of the DOb-based RRC.

**Figure 14:**
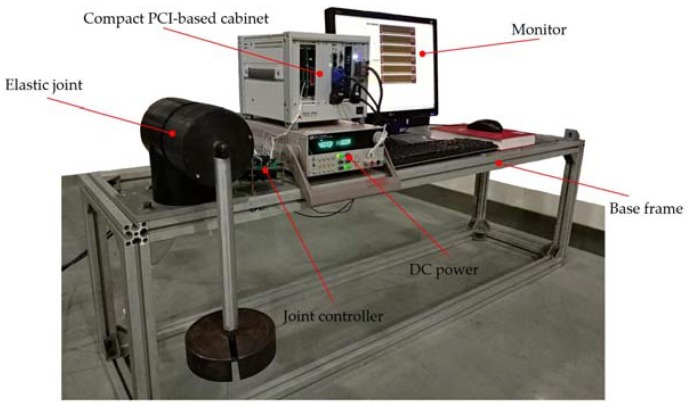
Experimental platform of the designed elastic joint.

Secondly, to validate the performance of the modular elastic joint and the DOb-based RRC, an experimental platform was established as shown in Figure 14. This platform mainly consisted of six parts, i.e., base frame, elastic joint with load, joint controller, Compact PCI-based cabinet, DC power and monitor. An input link of the elastic joint was fixed on the base frame and an output one held the load. All the actual parameters of the elastic joint were obtained by corresponding calibration experiments and listed in Table 1. From Table 1, it can be concluded that the designed elastic joint basically meets the design requirements.

**Figure 15:**
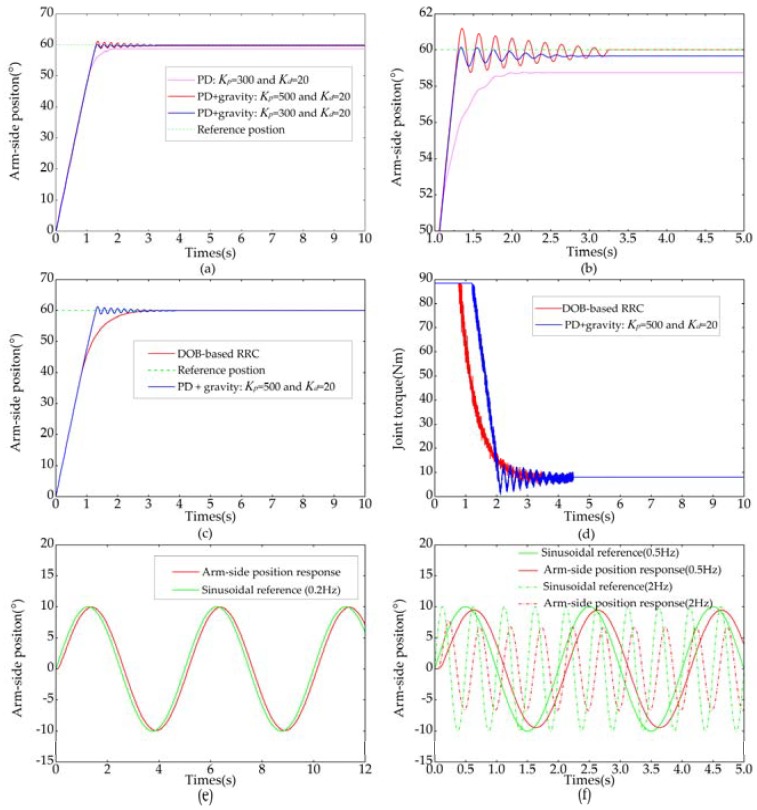
Experimental results of position control. (**a**) PD position control; (**b**) Magnified view of (**a**); (**c**) Comparison between PD control and RRC control; (**d**) Joint control torque; (**e**) Sinusoidal trajectory tracking under 0.2 Hz; (**f**) Sinusoidal trajectory tracking under different frequencies.

The position control experiments of the elastic joint were carried out with the same control parameters as in simulation and the results are shown in Figure 15. Figure 15a shows the step responses when conventional PD controller was employed. There was a steady-state error mainly due to arm-side load when PD controller was used. The steady-state error can be decreased if the gravity component of load is compensated. But, the steady-state error cannot be eliminated completely because the gravity component of load cannot be accurately estimated. Besides, uncertainties of friction, joint stiffness and external torque will result in the steady-state error. As it is shown in the figure, another way to reduce the steady-state error was to increase $K_{p}$. However, the stability deteriorated significantly. Figure 15c shows the comparison between PD control with gravity compensation and DOb-based RRC. When the position error was large, increasing rates of arm-side position in both control strategies were identical and basically equal to the maximum angular velocity of the elastic joint. Arm-side angular velocity fell rapidly in DOb-based RRC when the position error was small and arm-side position followed the reference position without vibration. Control torques of conventional PD controller and the proposed DOb-based RRC are shown in Figure 15d. The proposed DOb-based RRC avoided the oscillation of the control input. Figure 15e,f shows the trajectory tracking performances of the designed joint for sinusoidal references of different frequencies. It is clear from the Figure 15e that the proposed DOb-based RRC showed relatively high tracking performance. As shown in Figure 15f, the trajectory tracking performance deteriorates and the delay grew as the frequency increased, especially when the reference frequency was more than 2 Hz. It is easy to understand that the elasticity of the joint mainly answers for the delay and the DOb-based RRC for achieving vibration suppression will further pull down the system bandwidth. Experimental results show that the vibration can be suppressed by means of RRC and the excellent performance of the DOb-based RRC in point-to-point position control.

## 5. Conclusions

This paper presents the design of a compact modular rotary elastic joint for a humanoid manipulator. A customized torsional spring is designed based on FEM, which meets the performance requirements of admissible peak load, low stiffness, and compactness. The actual value of the spring stiffness, which was calibrated by means of experiment, was significantly higher than obtained by simulation. The difference between the actual and nominal properties of the material, the imperfections in the model used in the simulation and measurement errors probably led to the discrepancy. Fortunately, this discrepancy only makes the stiffness value of the elastic joint different from the expectation and has little effect on the performance of the elastic joint. The large central hole and fail-safe brake were respectively designed for cabling and power-off protection of the elastic joint. The designed elastic joint only used magnetic encoders for sensor information, which greatly reduces the cost. Employment of double-layer control structure in electrical system can reduce the burden on the controller and simplify system maintenance. Besides, the designed electrical system can be easily expanded to build a humanoid manipulator in future. The PPSeCo protocol employed in this paper for data communication between central controller and joint controller can ensure the transmission reliability, speed of data communication, and reduce noise in sensor signals. The specifications listed in Table 1 demonstrate the elastic joint suitable as a module for a humanoid manipulator.

This paper also proposed a high-performance robust point-to-point position control scheme for the designed elastic joint. Vibration of the elastic joint was suppressed by virtue of RRC, which can arbitrarily designate the ratio between the resonant and anti-resonant frequency according to the feedback of the nominal spring torsion. Moreover, robustness of the control system was achieved by using motor-side DOb and arm-side DOb.

The validity of the DOb-based RRC was demonstrated in the simulation results. Experimental results further showed the performances of the modular elastic joint and the efficacy of the controller. Design and control of a multi-DOF humanoid manipulator, which was equipped with the designed elastic joint, are the focus of our future work.

## Figures and Tables

**Table 1:** Design indexes and actual values of the elastic joint.

Parameters	Design Indexes	Actual Values
Maximum elastic output torque	≥60 Nm	67 Nm
Maximum angular velocity	≥0.6 rad/s	0.72 rad/s
Maximum diameter	≤120 mm	111 mm
Maximum length	≤150 mm	136 mm
Mass	≤2.5 Kg	2.4 Kg
Joint stiffness	≈350 Nm/rad	464.2 Nm/rad
Maximum angular range	360°	360°

**Table 2:** Control parameters of the simulation and experiment.

Parameters	Descriptions	Values
$J_{n}$	Nominal arm-side inertia	0.528 Kg·m^2^
$M_{n}$	Nominal motor-side inertia	1.567 Kg·m^2^
$K_{sn}$	Nominal joint stiffness	464.2 Nm/rad
$K_{p}$	Proportional gain	862
$K_{\upsilon }$	Differential gain	117.44
$K_{\Gamma }$	Feedback gain	7.58
$g_{m}$	Motor-side bandwidth	500 rad/s
$g_{l}$	Arm-side bandwidth	500 rad/s

## Abbreviations

The following abbreviations are used in this manuscript.

PPSeCo	Point-to-Point High Speed Serial Communication
VHDL	Very-High-Speed Integrated Circuit Hardware Description Language
FPGA	Field Programmable Gate Array
PCI	Peripheral Component Interconnect
DSP	Digital Signal Processor
MFLOPS	Million Floating-point Operations per Second
